# Thanks to our peer reviewers

**DOI:** 10.1002/lrh2.10289

**Published:** 2021-09-26

**Authors:** Nancy Allee

The publication of Issue 4 marks the completion of **Volume 5** of *Learning Health Systems*. An open access publication, the journal has advanced research and scholarship on learning health systems in partnership with our reviewers. With indexing in multiple major sources, we believe the journal is now on a sustainable, positive trajectory.

The journal has also published five Special Issues: “Patient Empowerment and the Learning Health System” (v.1); “Ethical, Legal, and Social Implications of Learning Health Systems” (v.2); “Learning Health Systems: Connecting Research To Practice Worldwide” (v.3); “Human Phenomics and the Learning Health System” (v.4); and “Collaborative Learning Health Systems: Science and Practice” (v.5). Our talented guest editors have been instrumental in helping these Special Issues come to fruition.

We are keenly aware that these achievements would not have happened without the dedicated efforts and insightful comments of all those individuals who accepted invitations to review submitted articles. With busy schedules and full commitments in the midst of the Covid‐19 pandemic, these individuals found the time and energy to contribute their expertise to our authors to help ensure that their papers met (and often exceeded) the journal's high standards for publication.

Please accept our sincere gratitude for your outstanding efforts.

*Charles P. Friedman*, Editor in Chief.

## REVIEWERS FOR VOLUME 5 LHS JOURNAL

The following list also includes reviewers of rejected manuscripts.

Julia Adler‐Milstein (United States)

John Ainsworth (United Kingdom of Great Britain and Northern Ireland)

Jill Allison (Canada)

Holt Anderson (United States)

Anna Andreasson (Sweden)

Patrick Archambault (Canada)

Panagiotis Balatsoukas (United Kingdom of Great Britain and Northern Ireland)

Maren Batalden (United States)

Marc Berger (United States)

Jeff Brown (United States)

Julie Bynum (United States)

Elizabeth Cohn (United States)

Heather Colvin (United States)

Derek Corrigan (Ireland)

Benoit Cossette (Canada)

Mary Costello (United States)

Susan Cronin (United States)

Jennie David (United States)

Brendan Delaney (United Kingdom of Great Britain and Northern Ireland)

Jordan Everson (United States)

Rhonda Facile (United States)

Marcy Fitz‐Randolph (United States)

Thomas Foley (United Kingdom of Great Britain and Northern Ireland)

Masanori Fukushima (Japan)

Sarah Greene (United States)

W. Ed Hammond (United States)

Lamiece Hassan (United Kingdom of Great Britain and Northern Ireland)

Frank Hulstaert (Belgium)

Michael Ibara (United States)

Amy Kilbourne (United States)

Harold Lehmann (United States)

Leslie Lenert (United States)

David McCallie (United States)

Loretta McCormick (Canada)

Marianne McPherson (United States)

Subha Madhavan (United States)

Laura Merson (United Kingdom of Great Britain and Northern Ireland)

Stephanie Morain (United States)

Jessica Moreau (United States)

Mark Musen (United States)

Eileen Navarro (United States)

Sally Okun (United States)

Nancy Pandhi (United States)

Richard Platt (United States)

David Purcell (United States)

Rohit Ramaswamy (United States)

Barbara Rath (United States)

Joshua Rubin (United States)

Elin Salmiranta (Sweden)

Edgar Schein (United States)

Madeline Schmitt (United States)

Michael Seid (United States)

Archana Tapuria (United Kingdom of Great Britain and Northern Ireland)

Jody Traver (United States)

Robert Verheij (Netherlands)

Jim Walker (United States)

Chris Webb (United States)

John Wilbanks (United States)

David Williams (United States)

Tracy Ziolek (United States)

